# PECVD of Hexamethyldisiloxane Coatings Using Extremely Asymmetric Capacitive RF Discharge

**DOI:** 10.3390/ma13092147

**Published:** 2020-05-06

**Authors:** Žiga Gosar, Janez Kovač, Denis Đonlagić, Simon Pevec, Gregor Primc, Ita Junkar, Alenka Vesel, Rok Zaplotnik

**Affiliations:** 1 Elvez Ltd., Ulica Antona Tomšiča 35, 1294 Višnja Gora, Slovenia; Ziga.Gosar@elvez.si; 2 Jozef Stefan International Postgraduate School, Jamova cesta 39, 1000 Ljubljana, Slovenia; 3 Department of Surface Engineering, Jozef Stefan Institute, Jamova cesta 39, 1000 Ljubljana, Slovenia; janez.kovac@ijs.si (J.K.); gregor.primc@ijs.si (G.P.); ita.junkar@ijs.si (I.J.); alenka.vesel@ijs.si (A.V.); 4 Faculty of Electrical Engineering and Computer Science, University of Maribor, Koroška Cesta 46, 2000 Maribor, Slovenia; denis.donlagic@um.si (D.Đ.); simon.pevec@um.si (S.P.); 5 Plasmadis Ltd., Teslova ulica 30, 1000 Ljubljana, Slovenia

**Keywords:** PECVD, HMDSO, PDMS, extremely asymmetric CCP, OES, XPS depth profiling, dust particles

## Abstract

An extremely asymmetric low-pressure discharge was used to study the composition of thin films prepared by PECVD using HMDSO as a precursor. The metallic chamber was grounded, while the powered electrode was connected to an RF generator. The ratio between the surface area of the powered and grounded electrode was about 0.03. Plasma and thin films were characterised by optical spectroscopy and XPS depth profiling, respectively. Dense luminous plasma expanded about 1 cm from the powered electrode while a visually uniform diffusing plasma of low luminosity occupied the entire volume of the discharge chamber. Experiments were performed at HMDSO partial pressure of 10 Pa and various oxygen partial pressures. At low discharge power and small oxygen concentration, a rather uniform film was deposited at different treatment times up to a minute. In these conditions, the film composition depended on both parameters. At high powers and oxygen partial pressures, the films exhibited rather unusual behaviour since the depletion of carbon was observed at prolonged deposition times. The results were explained by spontaneous changing of plasma parameters, which was in turn explained by the formation of dust in the gas phase and corresponding interaction of plasma radicals with dust particles.

## 1. Introduction

**Figure 1:**
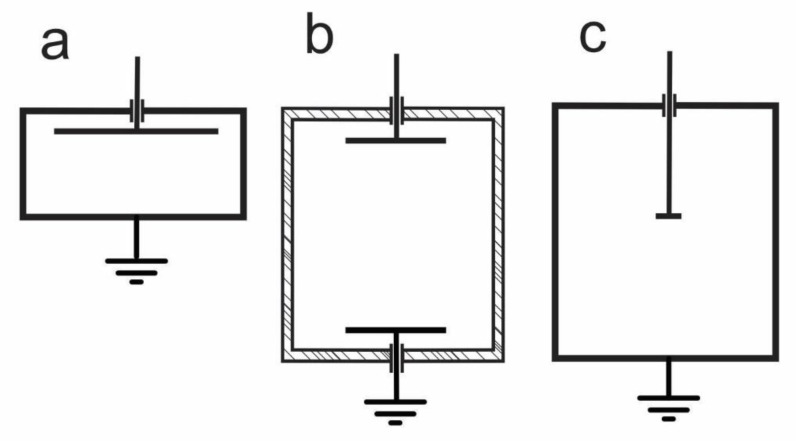
Three configurations of RF discharges sustained in capacitive mode: (**a**) moderately asymmetric; (**b**) symmetric; and (**c**) extremely asymmetric.

Plasma-enhanced chemical vapour deposition (PECVD) can be used for deposition of polymer-like silicon-containing coatings such as polydimethylsiloxane (PDMS) coatings. For deposition of such coatings, various precursors [1] can be used. Often the precursor is hexamethyldisiloxane (HMDSO) [2,3,4]. Gaseous plasma is sustained by various discharges, and a commonly applied one is a radio-frequency (RF) discharge where the RF power is applied in capacitive mode, i.e., the RF voltage is applied to a couple of electrodes. One electrode is usually just the grounded housing of a vacuum chamber, while the powered electrode is immersed into the metallic vacuum chamber using electrically-insulated feedthrough. Both electrodes are usually of large surface area. The powered electrode is often mounted into the vacuum chamber in such a way that the distance to the grounded housing is low enough to enable rather uniform plasma in the entire volume of the plasma chamber except in the gap between the powered electrode and the housing. The breakdown follows the Paschen curve, which has the minimum at roughly 1 cm torr [5]. The pressure inside the plasma chamber is often around 10 Pa, so the breakdown is likely to occur in the chamber volume rather than in the gap, providing the gap thickness is less than a few cm. An alternative is a discharge chamber with two electrodes of same dimensions mounted symmetrically in the chamber. The chamber can be metallic or made from a dielectric material with a couple of circular electrodes of equal dimensions inserted into the chamber. The configurations are schematically presented in Figure 1a,b. These configurations have been used by several authors. 

Ropcke et al. [6] used symmetric RF discharge (Figure 1b) operating at the standard industrial frequency of 13.56 MHz. The diameter of both electrodes was 13 cm, and the distance between the electrodes was 4 cm. They studied plasma properties using optical emission spectroscopy. Although the configuration was symmetric, they found some variation of the plasma parameters in the gap between the electrodes. The ratio between hydrogen emission lines H_α_/H_β_ was somewhat independent of the distance between the electrodes, indicating a rather uniform electron temperature in the entire volume between the electrodes. In contrast, the gas temperature as deduced from Doppler line broadening was almost 2000 K close to the powered electrode and below 500 K next to the grounded electrode.

Li et al. [7] used an asymmetrical capacitive discharge (Figure 1a) at 13.56 MHz and RF power up to 200 W. Plasma was sustained in the pulsed mode in a metallic reactor of volume 20 L at pressures between 10 and 100 Pa. Plasma was characterised by IR absorption spectroscopy and mass spectrometry, whereas the film thickness by ellipsometry. The reactor was filled with HMDSO, and its fragmentation was evaluated versus discharge time. The differences in the fragmentation degree clearly influenced the quality of the deposited film. As long as the molecular fragmentation was low, the structure of the films deposited on the powered electrode and the floating substrates was rich in an organic component. The concentration of this component in the films decreased with increasing fragmentation of the precursor in the gas phase. The difference, however, was not dramatic. The deposition rate, on the other hand, increased monotonously with increasing fragmentation in the gas phase, indicating a rather poor sticking of large molecules on the substrates. The deposition rate at the powered electrode was over an order of magnitude larger than on the floating substrates, which was explained by gradients of plasma density next to the powered electrode: more intensive fragmentation occurred next to the powered electrode while fragmentation in the positive column of plasma far from the sheath was not complete.

An asymmetric RF discharge was also used by Naddaf et al. [8]. They employed a hollow electrode and powered it with an RF generator operated at 13.56 MHz and power up to 300 W. The plasma density of the order of 10^17^ m^−3^ was estimated with a Langmuir probe. Both the deposition rate and Si–O–Si bond in the deposited film increased with increasing RF power at the constant pressure of 3 Pa. Hydrogen de-trapping was observed at higher power where the deposited film contained less Si–CH_3_ bonds as well. Increasing the precursor flow rate at constant pressure and power caused a significant increase of the film thickness indicating substantial consumption of the precursor for growth of the solid film at low flow rates (higher gas residence time). 

A moderately asymmetric RF discharge in capacitive mode was applied by Garofano et al. [9]. The powered electrode was of diameter 10 cm, the grounded of diameter 12 cm and the metallic chamber was grounded, too. The background gas was argon with a small admixture of Ne, Kr and Xe, and the HMDSO was injected in pulses. The pressure was between 5 and 6 Pa. At the RF power of 30 W, the self-bias voltage on the powered electrode was about −550 V. Plasma was characterised by optical emission spectroscopy. In such experimental conditions, dust appeared in the plasma reactor and peaked within a minute after injecting the precursor. 

Santos et al. [10] employed a similar reactor as Garofano to study the deposition kinetics of thin PDMS films using an admixture of 70% HMDSO, 10% Ar and 20% O_2_. The electrodes were made from stainless steel, and the films were deposited primarily onto the powered electrode. The total gas pressure was 8 Pa and the deposition time half an hour. The RF power was varied between 15 and 75 W. The reported self-bias was several 10 V, and it increased almost linearly with increasing RF power. The film thickness was almost perfectly linear with the reported self-bias. The increasing thickness was explained with increasing electron density in the gap between the electrodes, which was 3.5 cm. Increasing the power beyond 75 W resulted in the detachment of the films from the substrates, which was explained by an increase in the intrinsic stress with increasing film thickness. Pre-cleaning of the substrates with plasma treatment was found beneficial for adhesion of the coating. The relative concentration of C, Si and O in the solid film as deduced from XPS measurements did not depend on the RF power significantly. 

A similar moderately asymmetric configuration in the reactor of volume 0.1 m^3^ was also applied by Corbella et al. [11]. A round electrode of diameter 30 cm was powered with an RF generator at 13.56 MHz and powers 160 and 200 W. Argon was added to HMDSO to enhance the monomer fragmentation and stabilise the discharge. Unlike other authors, they also reported the gas residence time, which was about 1 s. The key parameter governing the quality of the films produced at that condition was found to be input power per gas flow.

Fanelli et al. [12] also employed the moderately asymmetric configuration. The 13.56 MHz generator was used for powering a stainless-steel plate. Various mixtures of argon, HMDSO and O_2_ were used, but the total pressure was kept almost constant at 27 Pa. A liquid-nitrogen-cooled trap was used to collect the products of plasma-induced fragmentation of the precursor. As many as 20 compounds were detected upon heating of the trap after sampling the fragments for 20 min. The predominant fragments observed at the selected conditions were tetramethylsilane and octamethyltrisiloxane. They found Si–O bond cleavage in HMDSO an important reaction at various conditions. Furthermore, they provided solid proof that oligomerisation occurred at those conditions. About 20% of HMDSO was lost upon plasma-induced fragmentation, and this value was independent of the concentration of oxygen in the original gas mixture. Oligomerisation was also observed by mass spectrometry by Alexander et al. [13]. They observed molecular oligomerisation of positively charged gaseous species at a rather low discharge power, whereas at large powers fragmentation prevailed. 

Plasma sustained by capacitive discharges is famous for the spontaneous formation of dust particles [14]. The plasma reactor was powered with a standard 13.56 MHz RF generator coupled in moderately asymmetric mode. Pulsed plasma was created in mixtures of argon and HMDSO. The formation of dust particles in gaseous plasma was observed in about one minute. With prolonged plasma duration, the dust particles disappeared from the gas phase, which was explained by ion drag forces. The dust was deposited on all surfaces facing plasma after turning off the discharge. The authors also discussed the sputtering of the organosilicon coating due to large self-biasing of the substrate, which was placed onto the powered electrode. 

The above-cited authors used either an almost symmetric coupling or moderately asymmetric coupling of the RF discharge in the capacitive mode (Figure 1a,b). Various discharge parameters were used by different authors, thus it is difficult to compare their results. However, it is possible to deduce qualitatively that the organic component in the deposited films decreased with increasing molecular fragmentation (discharge power) and with increasing oxygen admixture to the HMDSO or a mixture of HMDSO and a noble gas. Very little work, however, was reported on the behaviour of plasma and properties of deposited films for the case of extremely asymmetric coupling. Such a coupling may be interesting from both scientific and technological points of view. Although it is rarely mentioned in scientific literature, the symmetric (or moderately asymmetric) configuration of capacitive RF discharge has a technological drawback: the deposition of thin films from gaseous precursors occurs predominantly on the electrodes. This is because the electron heating occurs predominantly within the volume next to the electrodes. The samples that are kept away from the electrodes at the floating potential receive much less deposit than any sample placed onto an electrode. This effect might be suppressed using small electrodes. The properties of thin films deposited onto samples kept at floating potential are shown in this paper. Possible explanations of rather unexpected results are also provided. The objective of this paper is therefore a feasibility study on using extremely asymmetric capacitive coupled RF discharge for depositing thin films by PECVD method. 

## 2. Materials and Methods

Three configurations of RF discharges sustained in capacitive mode are schematically presented in Figure 1. First two configurations (Figure 1a,b), show a moderately asymmetric and symmetric configuration, respectively. These configurations are commonly used, as already mentioned in the Introduction. In the symmetric configuration (Figure 1b), both electrodes are of the same size, and the discharge tube is made from a dielectric—often glass. The electrons are heated in the volume next to the electrodes where the oscillating electric field is large. The electric field is marginal in the volume far from the electrodes and therefore only diffusing plasma is sustained in the majority of the discharge chamber volume. The heating of electrons is the same next to both electrodes. Both electrodes assume the same DC self-biasing, and the deposition rates are the same on both electrodes. 

In the case of moderately asymmetric RF discharge (Figure 1a), the area of the powered electrode is smaller than the grounded electrode. As a result, the DC self-biasing occurs practically only on the powered electrode. Any sample placed onto the powered electrode is thus subjected to bombardment by energetic positively charged ions. On the other hand, the DC self-biasing of the large grounded electrode is much smaller than on the biased electrode, so kinetic effects caused by ion bombardment are much less pronounced than in the case of the symmetric coupling. Many experimental and most industrial devices for deposition of thin films using the PECVD technique employ the moderately asymmetric configuration. The objects to be coated with a thin film are often placed into the entire volume of the discharge chamber where diffusing plasma is sustained. The objects are often placed onto a planetarium and rotated upon plasma treatment to suppress negative effects of any gradients of gaseous radicals. The moderately asymmetric configuration still suffers from the loss of radicals on the powered electrode where the deposition rate is often ten times larger than on the objects kept within the diffusing plasma at a floating potential. 

Figure 1c represents a case of extremely asymmetric configurations. By our definition, such a configuration is characterised by a large ratio between the area of the grounded and powered electrodes, often several 10-times. In this paper, we define extremely asymmetric RF discharge in a capacitive mode as a configuration where the ratio between the grounded and powered electrode areas is over 20. In such a configuration, the DC self-biasing of the grounded electrode is negligible, so any object placed onto the grounded electrode or on a planetarium in the volume filled with diffusing plasma receives almost the same flux of radicals (providing there are no large gradients in radical density within diffusing plasma). A small powered electrode should represent a small sink for plasma radicals since the loss rate is a product of the radical flux, sticking probability and surface area.

**Figure 2:**
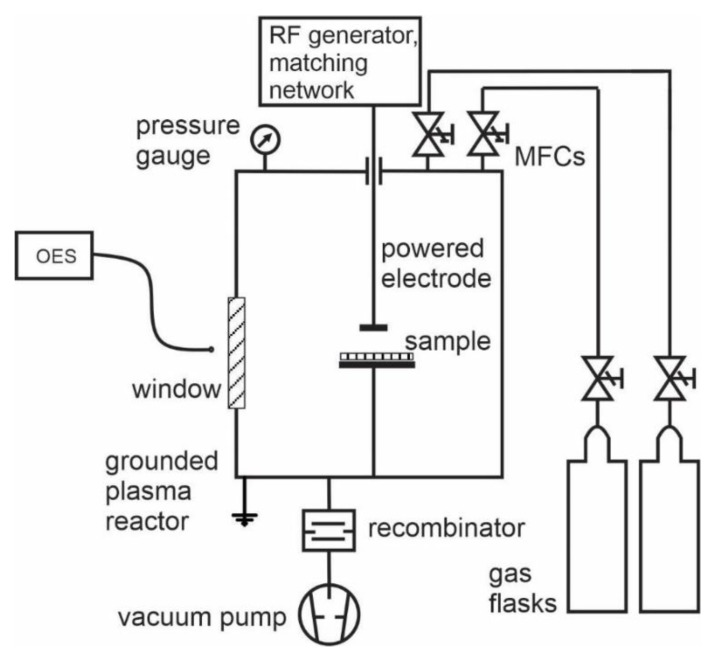
Schematic of the experimental system.

Experiments in this study were performed in a plasma reactor using the extremely asymmetric coupling of an RF generator, as shown schematically in Figure 1c. The experimental setup is shown in Figure 2. 

The reactor was made from stainless steel. It was pumped with a two-stage rotary pump of nominal pumping speed 80 m^3^/h. A trap made from nickel network was placed between the discharge chamber and the vacuum pump in order to assure for at least partial recombination of gaseous radicals and adsorption of molecular fragments and thus prevent such species enter the pump. The pressure was measured with an absolute vacuum gauge of pressure range 1–1000 Pa. Gases were introduced into the chamber via flow controllers calibrated for corresponding gases. Both HMDSO and oxygen were introduced at different rates in order to assure for desired gas mixtures. The total surface of the grounded walls was estimated to about 0.41 m^2^. The powered electrode was a small disk on a stainless-steel rod with a surface of approximately 1.5 dm^2^. The ratio between the area of the powered and grounded electrode was therefore about 0.03, thus assuring for extremely asymmetric conditions shown schematically in Figure 1c. 

The RF generator operated at the standard industrial frequency of 13.56 MHz and variable output powers up to about 1000 W. In such conditions, the sheath between gaseous plasma and the grounded housing was practically identical to a sheath that forms spontaneously on any floating object. The difference between space and floating potentials is, therefore, easily estimated from the electron temperature, as explained in classical literature [15]. The powered electrode assumed DC self-biasing, as explained in classical literature [15]. The DC self-biasing is roughly the same as the effective voltage of the power supply, which in our case is about 600 V. The sheath next to the powered electrode is not only characterised by a large oscillating bias but also expands much further from the surface as compared to the sheath next to grounded housing. The oscillating sheath of luminous plasma next to the powered electrode was visible with a naked eye, and its thickness was about 1 cm. Optical spectra were acquired from the plasma away from the sheath. We used a 0.5 nm resolution optical spectrometer Avantes AvaSpec 3648 (Avantes, Apeldoorn, The Netherlands) spectrometer that works over the range of 200–1100 nm, connected to an optical fibre. The lenses on the other side of the fibre were mounted in such a way that we sampled optical spectra from plasma close to substrates. These were placed onto the grounded housing, as shown in Figure 2. The optical window mounted on one flange was made from Pyrex glass which absorbs radiation in the UV range below about 320 nm. The substrates were made from aluminium. A commercial Al sheet of thickness 0.3 mm was cut to rectangular pieces of about 1 cm × 1 cm. The samples were roughly cleaned with ethanol before insertion into the plasma reactor. No polishing was applied. A part of each sample was covered with an adhesive tape which was carefully removed after performing plasma experiments. The step formed between the uncovered and covered parts of a substrate was then probed by atomic force microscopy (AFM, NT-MDT Spectrum Instruments, Moscow, Russia) to estimate the thickness of the deposited film. This thickness was then used for calibration of depth profiles which were acquired by XPS.

The samples were placed into the reactor, which was then pumped for a few minutes until the pressure, as measured with the gauge, stabilised at 1.7 ± 0.2 Pa. Then, gases were introduced upon continuous pumping, and the discharge was turned on for an appropriate period. Treatment times were up to 1 min. The uniformity of the coatings was checked with a scanning electron microscope (SEM, JEOL, Tokio, Japan). A typical SEM image of an HMDSO coated sample is presented in Supplementary Materials (Figure S1).

Samples were characterised by X-ray photoelectron spectroscopy (XPS). A TFA XPS instrument from Physical Electronics (Chanhassen, MN, USA) was used with the base pressure in the analysis chamber of about 6∙10^−10^ mbar. The samples were excited with X-rays over a specific 400 µm area using monochromatic Al K_α1,2_ radiation at 1486.6 eV. For the photoelectron detection, a hemispherical analyser, positioned at an angle of 45° with respect to the surface normal, was used. The energy resolution was about 0.6 eV. The measured spectra were analysed using MultiPak v7.3.1 software from Physical Electronics, which was supplied with the spectrometer. Ar-ion sputtering during XPS depth profiling was performed with the Ar-ions of energy of 4 keV. Ar sputtering with monoatomic gas may induce preferential sputtering and changes in surface chemistry, in particular for organic films. Usually, the preferential sputtering of O-atoms is present during profiling of organic films containing oxygen species. For inorganic SiO_2_-like films, the change in composition during XPS depth profiling due to preferential sputtering is much smaller. From both mentioned effects we expect that for our films, which are hybrid inorganic–organic C–Si–O films, probably a smaller extent of preferential sputtering of oxygen may be expected. This might be relevant for the depth of films, where an organic component was still present, i.e., at the film/substrate interface. Regardless, we expect that preferential sputtering of oxygen in this region will cause a relative reduction of O for about 10% of the reported atomic concentration of oxygen, which is not so relevant for our results. In depth profiles, the Ar sputtering time is replaced with depth even though the sputtering rates are not the same for Al-oxide and SiOCH films. However, the rough approximation about similar sputtering rates [16] is still good enough for the subject of this study, which is mainly a comparison of thicknesses and composition of the SiOCH films and less related to the thickness of the Al-oxide layer covering the Al substrate.

## 3. Results and Discussion

### 3.1. Optical Spectra

**Figure 3:**
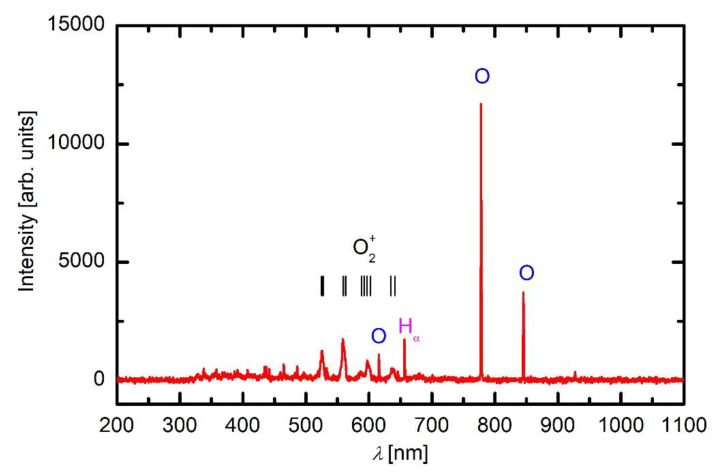
A spectrum of plasma in oxygen at 10 Pa and 200 W.

**Figure 4:**
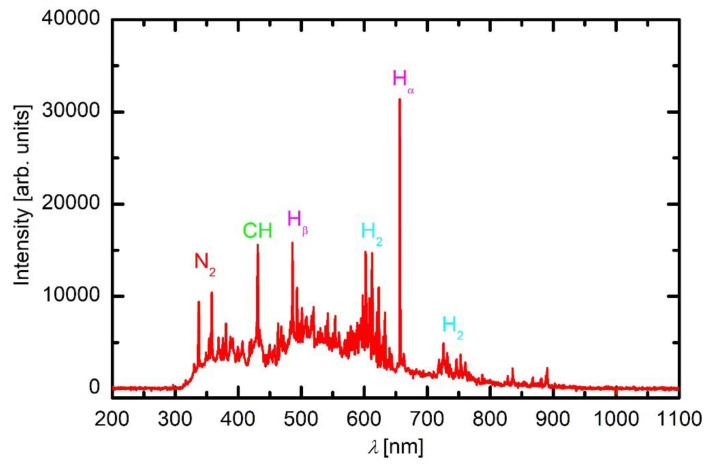
A spectrum of plasma in HMDSO at 10 Pa and 200 W.

**Figure 5:**
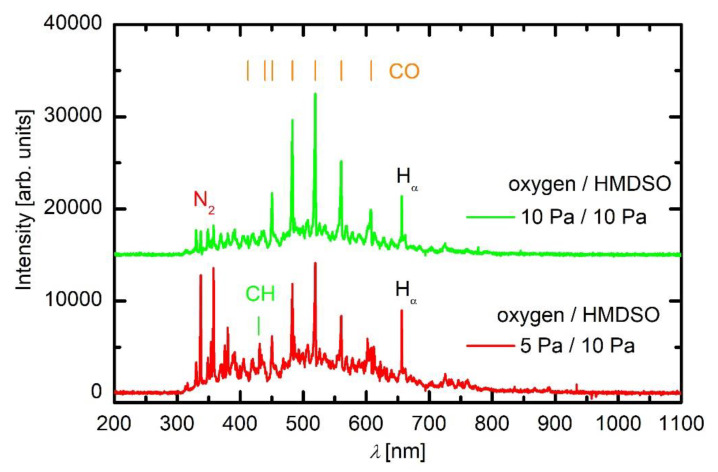
A spectrum of plasma in oxygen/HMDSO mixture 1/2 (bottom curve) and 1/1 (top curve) at 200 W.

Plasma was characterised by optical emission spectroscopy. Typical spectra are shown in Figure 3, Figure 4 and Figure 5. Figure 3 represents an optical spectrum of plasma sustained in oxygen.

The predominant peaks at about 777 and 845 nm correspond to radiative transitions of neutral oxygen atoms. Apart from these ones can observe other features such as H_α_ line at 656 nm, and O_2_^+^ bands in the range from about 500 to 650 nm which arise from radiative transitions of ionised oxygen molecule [17]. The appearance of hydrogen atomic line is typical for gaseous plasma sustained in chambers which have never been baked and are pumped by fore pumps only. Namely, the residual atmosphere in such vacuum systems usually contains water vapour which dissociates to OH and H radicals upon plasma conditions. The OH band which should appear at the bandhead of 309 nm is not visible in the spectrum due to poor transmission of the glass window in the UV range. There are some weak spectral features also in the range from about 320 and 550 that could be attributed to CO bands. The CO is a product of an interaction between O-atoms and O_2_^+^ ions or both with any carbon in the system. The origin of carbon is in the deposited films. Namely, the spectra were acquired after performing numerous experiments with HMDSO. Upon these experiments, a layer containing Si, O, C, and H were deposited on the walls, and the carbon is slowly etched when such layers are exposed to oxygen plasma. 

An optical spectrum acquired in HMDSO plasma is shown in Figure 4. The spectrum is completely different from that acquired in oxygen. The O lines are not distinguishable, and the highest peak is H_α_. Apart from the dissociation of water vapour, the atomic hydrogen appears as a result of dissociation of both precursor molecules and their fragments. There are numerous other bands and lines in the visible range which form a virtual continuum, probably because of overlapping of numerous molecular bands. The significant lines were identified and are marked in Figure 4. Among them, the molecular hydrogen bands (Fulcher bands) are most extensive. Such intensive bands indicate a qualitatively large concentration of hydrogen molecules in gaseous plasma created in HMDSO. 

The origin of hydrogen molecules in gaseous plasma of hydrocarbons sustained at low pressure is usually attributed to the surface association of H atoms to H_2_ molecules. A clearly distinguished CH band with the bandhead at 431 nm indicates another channel for the radicalisation of the precursor molecule. At electron impact, methyl radicals are likely to be formed [18], and these radicals are further dissociated upon plasma conditions to form CH radicals. Although the sticking coefficient for CH on any surface kept at room or slightly elevated temperature is very large [19], their production in the gas phase is obviously large enough to identify the bandhead doubtlessly. The nitrogen band is probably due to slight leakage of the system, but the broad virtual continuum expanding over the entire visible range could be attributed to various effects. Both CO molecules, C_2_ dimers and H_2_ molecules radiate in the visible part of the optical spectrum, so any distinguishing is regarded as impossible. However, the spectrum presented in Figure 4 indicates a number of atoms, molecules and radicals in plasma sustained in HMDSO and thus a variety of gas-phase dissociation reactions.

Figure 5 represents optical spectra of plasma sustained in the mixture of 2/3 HMDSO and 1/3 O_2_ (bottom curve) and 1/2 HMDSO and 1/2 O_2_ (top curve). Apart from the virtual continuum, which is still dominant in integral radiation in the visible range, the spectrum is much different from that in HMDSO plasma. The CH bandhead at 431 nm is suppressed, and so is the radiation arising from hydrogen molecules and atoms. The major radiation in this gas mixture arises from CO bands. The major “lines” arising from CO Angström system are marked in Figure 5. There are a couple of other systems (Asundi bands and the triplet bands) that partially overlap with the Angström system, but they are not marked in this figure for the sake of clarity. The production of CO molecules in the experimental plasma reactor is therefore extensive. The formation of the CO molecule upon a collision of oxygen radical with a carbon-containing molecule is highly improbable since the reaction is highly exothermic and would require a super-elastic collision to assure for the conservation of energy and momentum. The production of CO is therefore likely to occur on surfaces. As mentioned in the previous paragraph, the sticking coefficient of CH radicals on surfaces kept at room temperature is large. For CH_2_ radicals, it is lower but still significant. According to Tichmann [19], the sticking coefficient of low-energy CH_3_ radicals on a surface at room temperature is still about 0.5. The CH_x_ radicals (x < 4) therefore stick well to surfaces. Many radicals undergo surface association to light stable hydrocarbons that desorb from the surface, but many interact with atomic oxygen. The atomic oxygen interacts readily with such deposits and causes rapid removal of the deposited hydrocarbons so the deposited film does not contain a measurable amount of hydrogenated carbon, as becomes obvious from results of film characterisation presented below. The extensive CO bands, as observed in Figure 5, are therefore attributed to surface reactions.

Further addition of oxygen causes even more pronounced CO radiation, as shown in Figure 5 (top curve). The CH bandhead is hardly recognisable, and the entire spectrum is rich in CO radiation. The virtual continuum is further suppressed (as compared to Figure 4 and Figure 5, bottom curve), thus one can speculate that an important contribution of radiation to the virtual continuum is arising from radiative transitions of molecules other than CO radicals. Moreover, as mentioned above, quantification of radiation in the virtual continuum between about 300 and 700 nm is regarded as speculation. However, the predominant CO radiation indicates extensive oxidation of carbon-containing molecules.

**Figure 6:**
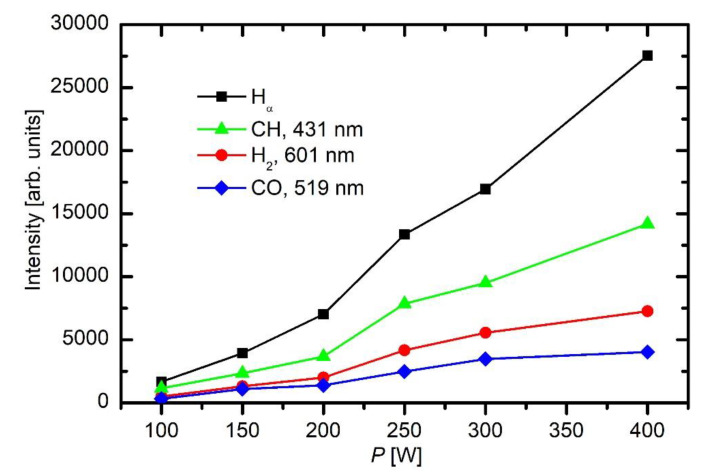
Intensities of some spectral features versus RF power in HMDSO plasma at 5 Pa.

Figure 6 represents the intensities of most prominent spectral features in HMDSO plasma versus discharge power. 

As expected, all spectral features increase with increasing power. This is explained by higher electron density and temperature at elevated powers. The differences between the lowest power (100 W) and highest (400 W) are huge. For example, the height of the H_α_ peak at 400 W is over an order of magnitude larger than at 100 W. 

### 3.2. XPS Characterisation

**Figure 7:**
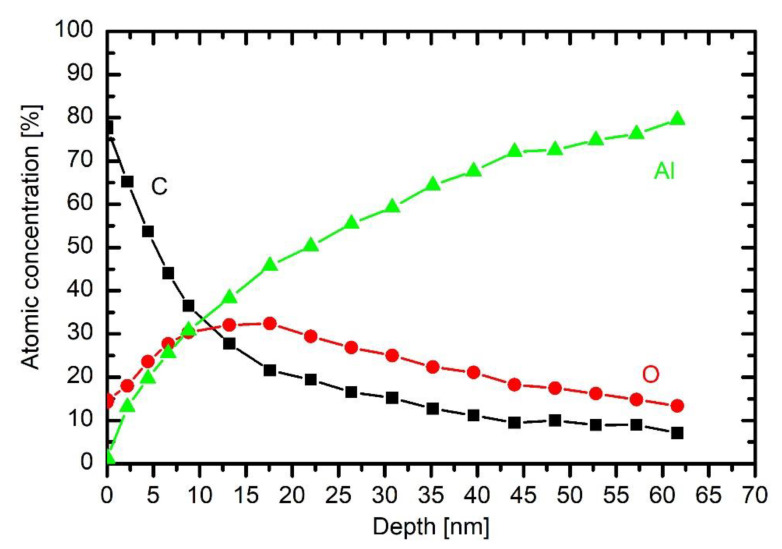
XPS depth profile of an untreated sample.

Samples were exposed to a gaseous plasma created in HMDSO with or without admixture of oxygen. After the treatment at different gas concentrations, discharge powers and treatment times, they were characterised by XPS. The depth profiles were acquired upon etching of the deposited films with Ar ions until the signal from the substrate prevailed. Figure 7 represents a typical depth profile of an untreated sample. 

The sample was taken as received, so the aluminium surface is oxidised. The oxygen concentration on the very surface is larger than Al concentration, but after about 10 min of Ar ions etching, the concentrations become equal. The concentration of oxygen slowly decreases with prolonged etching. Such a depth profile is typical for rough metals covered with an oxide film. The slow decrease of the oxygen concentration is explained by rich surface morphology rather than diffusing layer containing partially oxidised aluminium. On top of the oxide layer, there is a layer of organic impurities. The concentration of carbon in the interface between the oxide and bulk aluminium follows the concentration of oxygen, which is again typical for rough substrates. Carbon is therefore not present in the substrate but rather forms a surface film of organic impurities. 

#### 3.2.1. Influence of Oxygen Concentration in the Gas Mixture

**Figure 8:**
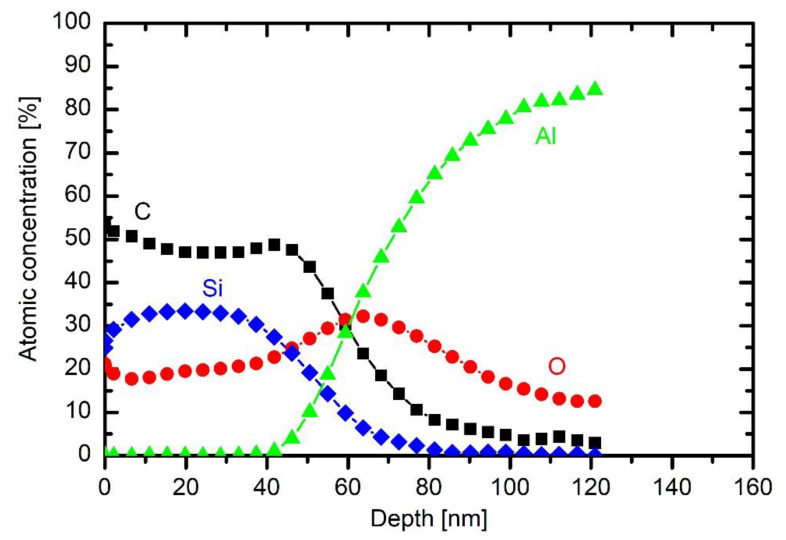
XPS depth profile of a sample exposed to plasma of 10 Pa HMDSO for 30 s at 200 W.

Figure 8 represents a depth profile of a sample treated in HMDSO plasma for half a minute. The broad interface between bulk aluminium and the coating is explained as for the untreated sample whose depth profile is in Figure 7. 

The broad maximum in the oxygen concentration peaking at a depth of about 60 nm is therefore explained by the persistence of the initial oxygen film on the as-received substrate. The coating contains about 50 at.% carbon, 30 at.% silicon and 20 at.% oxygen. Taking into account the accuracy of determination of atomic concentrations from XPS spectra acquired upon Ar ion sputtering, the coating’s composition is close to polydimethylsiloxane ((C_2_H_6_OSi)_n_). The different ratio between Si and O right on the surface observed practically on all samples could be attributed to the differences in the sputtering yields for Si and O with Ar ions (sputtering yield for oxygen is larger than for Si). The composition of the coating is rather uniform throughout the coating, which indicates little change of plasma parameters during the deposition process. 

**Figure 9:**
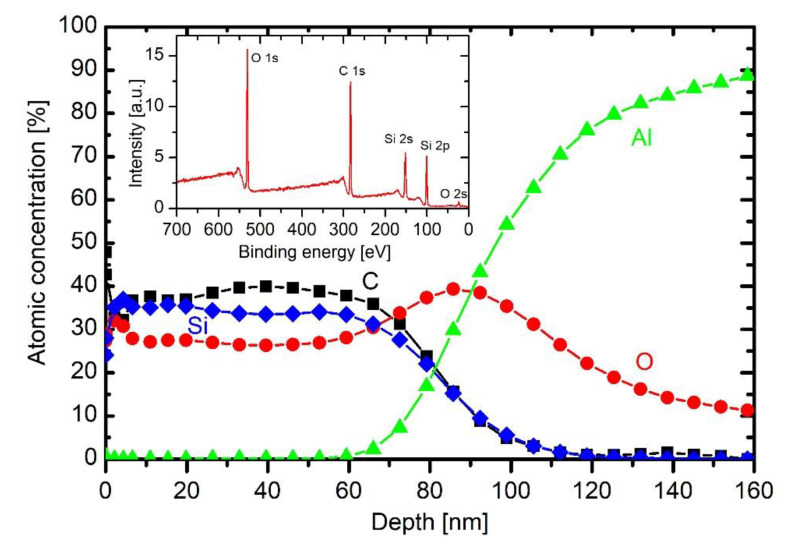
XPS depth profile of a sample exposed to plasma of 10 Pa HMDSO and 2.5 Pa O_2_ for 30 s at 200 W, with inserted wide energy range XPS spectrum obtained on the surface of this sample.

Addition of a small concentration of oxygen into HMDSO plasma causes depletion of the organic component in the coating, as deduced from the results in Figure 9. This effect is consistent with the results of optical emission. The concentration of about 20% oxygen in the gas mixture enables deposition of the coating with about 40 at.% of carbon, as revealed in Figure 9. The concentration of oxygen is still lower than silicon, but the difference is smaller than in the case of HMDSO plasma. The broad interface between the coating and the bulk aluminium is practically the same as in Figure 8. The same applies for interfaces in the depth profiles at larger oxygen concentrations in the original gas mixture. Figure 9 also shows a typical wide energy range XPS spectrum obtained on the surface of the sample. It contains peaks O 1s, C 1s, Si 2s, Si 2p and O 2s. No other elements were detected on the surface of this coating. 

The behaviour of carbon concentration at the interface indicates that the layer of organic impurities persists at the interface, although a substantial concentration of oxygen was added to the gas mixture. From this observation, one can conclude that even 43% oxygen in the gas mixture used for deposition of coatings does not cause significant oxidation of the organic impurities, which are presented on the surface of as-received substrates. Instead, they are buried at the interface by depositing the coating rich in silicon and oxygen. 

The concentration of 1/3 oxygen and 2/3 HMDSO causes a significant difference in the composition of the deposited coating. Next to the interface (i.e., between 40 and 70 nm), the composition is as expected, i.e., similar concentrations of Si and O and larger concentration of carbon. On top of this film, however, there is a coating depleted from carbon. Namely, the concentration of Si and O in the film between a few nm and about 25 nm is almost twice as the concentration of carbon. The discharge parameters (gas flows, power) remained the same throughout the deposition process, thus the broad minimum in the C concentration should be explained by changeable plasma parameters without changing the discharge conditions. 

This effect is even more pronounced for the case of 43% oxygen and 57% HMDSO. There is a thin film containing Si, O and C on the interface between the coating and the substrate, but the majority of the coating contains little carbon. The depth evolution of Si, O and C is similar to that of 1/3 oxygen and 2/3 HMDSO mixture, except that the depletion of the organic component is more pronounced in the case of 43% oxygen addition. The rather unexpected depletion can be explained with a progressive change of gas-phase or surface kinetics during the deposition. 

As revealed from the literature survey in the Introduction, the HMDSO plasma tends to develop clusters, often called oligomerisation [12]. These clusters grow with time and eventually form dust particles [9]. Once a critical size of particles is achieved, they become negatively charged due to attachment of slow plasma electrons. The negative charge of small particles prevents their contact to any walls of the plasma reactor (including substrates) due to repulsive electric filed in the sheath between the bulk plasma and the wall. The particles remain levitating in the plasma and keep growing as radicals stick to them. A variety of radicals form upon plasma conditions in mixtures of oxygen and HMDSO as confirmed by optical spectra. When the radicals collide with the particles, they may undergo exothermic reactions. Condensation itself is an exothermic event, but the released energy is marginal. On the other hand, there are surface reactions that dissipate significant energy upon interaction. Among them, heterogeneous surface recombination usually prevails in weakly ionised gaseous plasma. There are both oxygen and hydrogen atoms in plasma sustained in a mixture of oxygen and HMDSO, but O atoms are consumed extensively by surface chemical reactions that lead to the formation of CO radicals, as revealed in Figure 5. Namely, the O-atom peaks at 777 and 845 nm are hardly observable in Figure 5. Hydrogen atoms are observable in all plasma spectra presented in Figure 3, Figure 4 and Figure 5, while oxygen atoms definitely form in such plasma but are only clearly recognised in Figure 3 due to the consumption by chemical reactions. The energy released on a particle surface due to surface recombination is about half of the dissociation energy of parent molecules, i.e., about 2 eV per atom recombining on the surface. As a result, any particle levitating in plasma rich in H atoms warms up significantly. Upon steady conditions, the temperature of a particle levitating in plasma depends on the recombination coefficient. The coefficients have never been determined for dust formed in such complex plasma, so it is impossible to speculate about the temperature of the particles, but it is clear that the temperature increases with time. The recombination coefficients usually increase with increasing temperature [20]. Increasing temperature causes a dramatic increase in the oxidation rate of organic material upon treatment with oxygen atoms [21]. The final consequence of the interaction between oxygen atoms and particles is, therefore, extensive oxidation of the organic matter of the particle. In fact, the H_α_ peak in the optical spectrum of plasma rich in oxygen (Figure 5) is much smaller than peaks of the CO band, indicating qualitatively strong oxidation of organic component. The H_α_ peak in HMDSO plasma is much larger than any other spectral features (Figure 4). 

The rather unexpected depth profiles of 33% and 43% oxygen addition in gas mixtures can therefore be explained by the time evolution of the oxygen consumption in gaseous plasma. At the beginning of the deposition, there is practically no cluster in plasma so the oxygen, although present in the gas phase in rather large concentration, does not cause chemical reactions in the gas phase, so the coating contains a significant amount of the organic component. As particles appear and grow, more oxygen is used for oxidation of the organic component and the CO bands prevail in optical spectra. Carbon monoxide practically does not stick to surfaces, but the silica-rich particles do upon turning off the discharge. This could be a feasible explanation for the gradual decrease of the carbon concentration at prolonged deposition time. The effect is more pronounced at larger oxygen concentration. 

The high concentration of carbon on the very surface (Figure 8 and Figure 9) is due to secondary contamination of samples on the way between the plasma lab and XPS characterisation. In fact, the concentration on the very surface is almost the same for all depth profiles.

**Figure 10:**
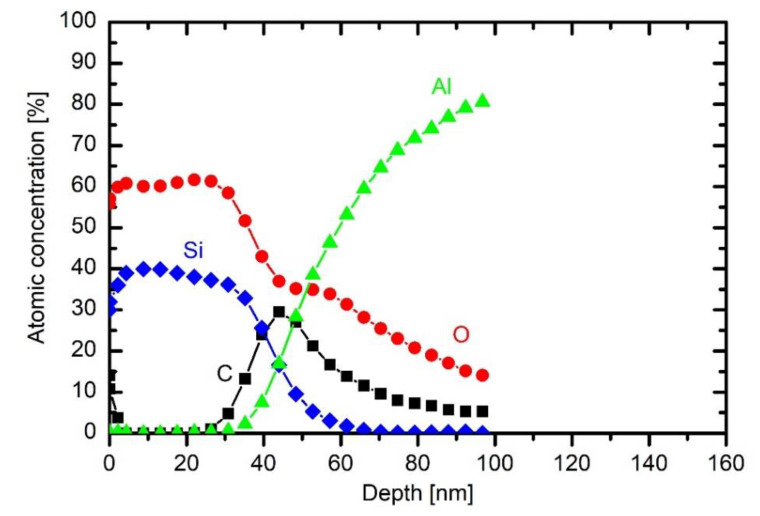
XPS depth profile of a sample exposed to plasma of 10 Pa HMDSO and 20 Pa O_2_ for 30 s at 200 W.

The coating obtained upon treatment of a sample in HMDSO with a large concentration of oxygen is completely different. Figure 10 shows a profile at oxygen/HMDSO mixture 2/1. 

The coating is free from carbon, and the ratio between oxygen and silicon is close to stoichiometric SiO_2_. The result is consistent with the observation reported by other authors [10]. The carbon at the interface between the substrate and the coating still persists; such oxygen atoms, although rather dense at these conditions, were not able to oxidise the impurity layer before it was buried by the deposit. Although the HMDSO is rich in carbon, the oxygen flux on the surface was obviously large enough to cause immediate oxidation of any organic compound that might have stuck on the surface upon film growth. The film thickness is much smaller than at lower oxygen concentration in the gas mixture due to the absence of an organic component. 

The extremely asymmetric capacitive discharge sustained by RF generator, therefore, enables deposition of different coatings, depending on the oxygen addition to the HMDSO precursors, but one should beware of temporal effects of oxygen consumption, as described above. 

#### 3.2.2. Influence of Discharge Power

**Figure 11:**
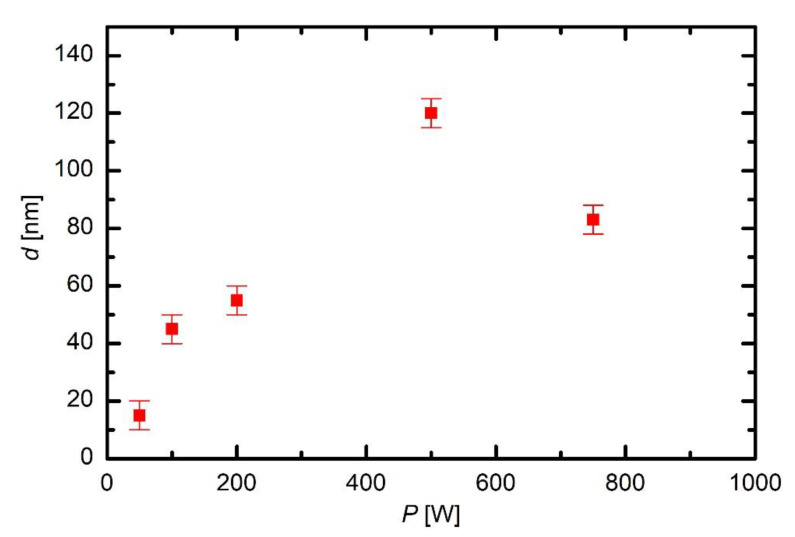
The thickness of coatings on samples versus the discharge power (30 s in HMDSO plasma at 10 Pa).

**Figure 12:**
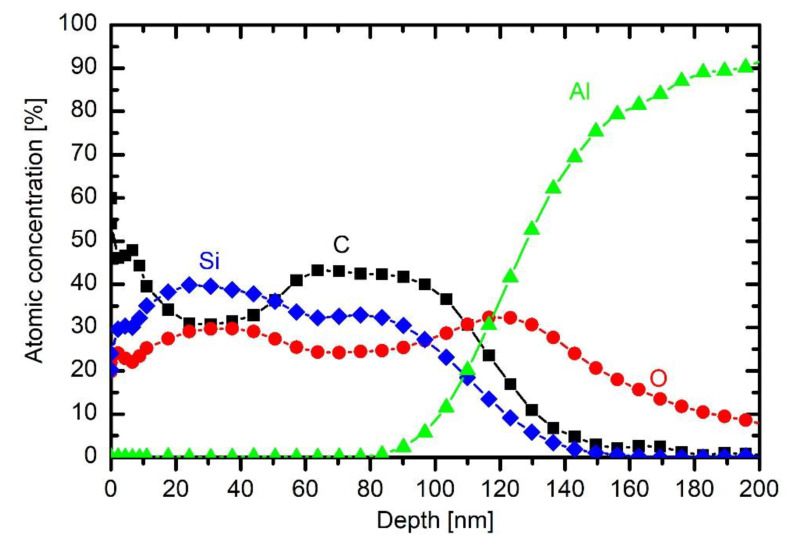
XPS depth profile of a sample exposed to plasma of HMDSO at 10 Pa for 30 s and 500 W.

**Figure 13:**
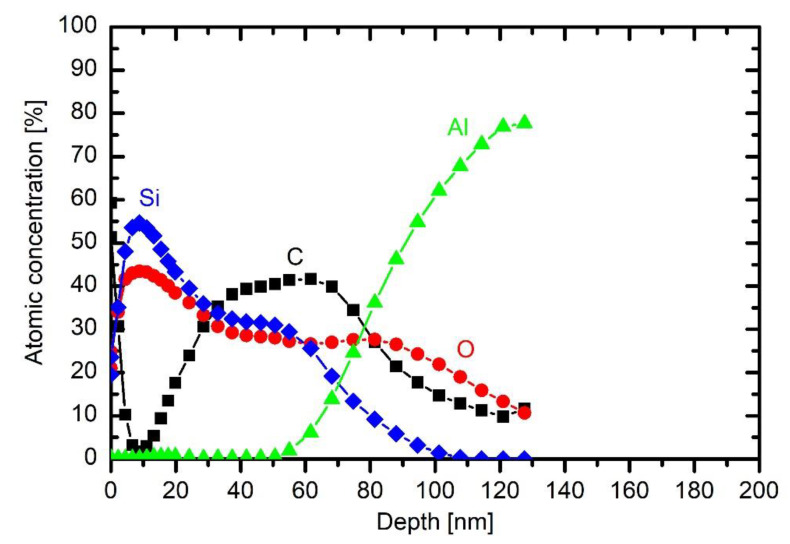
XPS depth profile of a sample exposed to plasma of HMDSO at 10 Pa for 30 s and 750 W.

The discharge power governs the plasma parameters, in particular the electron density, which in turn governs the gas-phase reactions and thus the fragmentation of the precursor. A set of experiments was performed at different discharge powers between 50 and 750 W with constant other parameters. The results of these coatings are presented in Figure 11, Figure 12 and Figure 13 and in Figure 8. 

Figure 11 represents the thickness of coatings versus discharge power. The coatings were deposited in 30 s of HMDSO plasma at 10 Pa. It can be observed that, at low powers, the thickness of coatings increases almost linearly with power. However, at higher power, the thickness starts to decrease. This can be explained by a closer inspection of the depth profiles.

The thickness of the coating of a sample treated at the lowest discharge power of 50 W is much smaller than at 200 W (Figure 8). The observation is explained by poor fragmentation of the precursor upon plasma conditions. As mentioned above, the extremely asymmetric discharge causes practically all available power absorbed in the oscillating sheath next to the powered electrode, so the concentration of electrons in bulk plasma is small. The distribution of carbon is similar to that of an untreated sample (Figure 7). There is some silicon on the surface, so even this very low discharge power enables deposition of a film rich in organic component, but the deposition rate as estimated from the XPS depth profile is below nm/s. 

A double discharge power enables the deposition of a thicker coating as revealed from the depth profile of a sample treated with HMDSO plasma at 100 W. Carbon prevails in the depth profile indicating a large fraction of organic component in the film. The result is similar to that reported by Naddaf [8] for the case of moderately asymmetric coupling. Aluminium persists right to the very surface so one could speculate that the coating at this power is not uniform enough to shade the substrate completely. Namely, the average thickness of the deposited film is several 10 nm. On the other hand, Si also persists deep from the surface so the speculation may not be correct. The original roughness of this sample at the spot sampled by XPS might be particularly large. 

The depth profile of a sample treated at 200 W is shown in Figure 8 and is discussed above. The depth profile at 500 W is shown in Figure 12. The coating is thicker than at lower powers, indicating that such a rather large power causes significant fragmentation, which is useful for more rapid deposition of the thin film. The composition of the coating on the substrate is somewhere in between those obtained at 200 W without oxygen and 200 W with 2.5 Pa oxygen: roughly 45 at.% C, 28 at.% O and 37 at.% Si. The increased power has therefore similar effect to the coating composition as the addition of a small amount of oxygen to the gas mixture. 

More pronounced is the effect at the largest power adopted in this work. Figure 13 presents the depth profile at 750 W and the same other conditions as in Figure 8 and Figure 12. The deposit on the substrate contains a significant fraction of organic compound, but, closer to the surface, the carbon is heavily depleted. The depth profile in Figure 13, therefore, resembles those of 33% and 43% oxygen addition in HMDSO. Thus, one can conclude that large discharge power results in a coating of similar properties as the addition of moderate amounts of oxygen to the gas mixture. The depletion of the organic compound at such a large discharge power in the extremely asymmetric coupling but in HMDSO plasma could be explained by different effects. The trivial one is the persistence of water vapour in the experimental reactor. As mentioned above, the reactor is only pumped with a rotary pump and never baked, thus the “base pressure” (i.e., the pressure achieved in the reactor before introducing precursor) is rather poor. The base pressure was approximately 1.7 Pa. The pressure increased to 10 Pa upon continuous introduction of the precursor (and also continuous pumping of the system). As mentioned above, the residual atmosphere contains mainly water vapour. The oxygen concentration in the reactor upon deposition of the coating is therefore much less than the concentration of carbon. Even if all oxygen from water vapour interacts with carbon from HMDSO, there should be excessive carbon in the system, thus the negligible concentration of carbon in the surface film down to about 20 nm in Figure 13 cannot be attributed to complete oxidation of carbon-containing materials. 

The poor concentration of carbon in the deposited film is instead explained by partial transformation of HMDSO to light hydrocarbons that are pumped away from the plasma reactor. The formation of methane, acetylene and ethylene has been already confirmed by residual gas analyses for the case of plasma sustained in a moderately asymmetric RF discharge [14,22,23]. The high power as adopted for the experiment whose result is shown in Figure 13 enables extensive fragmentation of precursor, so dust particles form quickly. The dust particles assume a high temperature so thermal decomposition cannot be neglected. As shown by Pressinger and Bruggemann [24], the thermal decomposition of HMDSO causes the formation of methane and ethane with some other light hydrocarbons. The remaining composition of hot clusters or dust particles contains silicon and oxygen. Due to the shortage of any other source of oxygen (water vapour), the particles retain a larger concentration of silicon than oxygen. As the discharge is turned off, the potential drop across the sheath between plasma and any surface vanishes so the particles rich in Si and O stick to the surface of the sample. The required condition for such an evolution of gas chemistry is oligomerisation, clustering and final evolution of dust particles. The reactions propagate with time and are faster at higher powers. This is a feasible explanation of why the Si-rich film appears only at high powers at given plasma treatment time and pressure (30 s and 10 Pa in Figure 11, Figure 12 and Figure 13). 

## 4. Conclusions

The extremely asymmetric electrode configuration in a low-pressure plasma reactor powered by an RF generator in capacitive mode was found to be useful for the deposition of various coatings using HMDSO with different admixtures of oxygen as source gases. Dense plasma in such a configuration is confined to an oscillating sheath next to the powered electrode and rather uniform diffusing plasma occupies the entire volume of the discharge chamber at the pressure of 10 Pa. The deposition rate and the composition of the coating depend on the discharge power and the gas composition. The organic fraction of the coating decreases dramatically with increasing power and increasing oxygen concentration at such discharge conditions. At low power and oxygen concentration, the coating is rather uniform, and its composition does not depend much on the treatment time. At large discharge power, the initial coating growing of a substrate resembles polydimethylsiloxane, but, for prolonged treatment, the composition changes and the organic component almost vanishes, although the discharge parameters remain constant. A similar effect was observed at increasing oxygen concentration. It was found that, in both cases, a feasible explanation for the observed carbon depletion of the film is the formation of clusters and dust particles, which heat upon plasma conditions due to exothermic surface reactions. The high temperature of the particles adds other channels for the loss of radicals and thus influences the growth of the coating. The surface reactions play an important role in the composition of both the particles in the gas phase and the coating. The heterogeneous surface recombination of hydrogen and oxygen atoms cause a thermal load, which was found particularly extensive for dust particles levitating in the gas phase. As the temperature of dust particles increase, the thermal decomposition becomes a significant source of light hydrocarbons that are pumped away from the system, so the coating at high power becomes almost free from carbon.

## Supplementary Materials

The following are available online at https://www.mdpi.com/1996-1944/13/9/2147/s1, Figure S1: SEM image of the HMDSO coating.
