# Supplementary material for: PECVD of Hexamethyldisiloxane Coatings Using Extremely Asymmetric Capacitive RF Discharge
Source: Materials (Basel). 2020 May 6;13(9):2147. doi: 10.3390/ma13092147 (PMC7254392; doi:10.3390/ma13092147)
Supplement: Supplementary file 1 [file materials-13-02147-s001.pdf]

materials

*Supporting Information*

# PECVD of Hexamethyldisiloxane Coatings Using Extremely Asymmetric Capacitive RF Discharge

**Žiga Gosar [1,2], Janez Kovač [3], Denis Đonlagić [4], Simon Pevec [4], Gregor Primc [3,5], Ita Junkar [3], Alenka Vesel [3,5] and Rok Zaplotnik [3,5,*]**

1 Elvez Ltd, Ulica Antona Tomšiča 35, 1294 Višnja Gora, Slovenia; Ziga.Gosar@elvez.si
2 Jozef Stefan International Postgraduate School, Jamova cesta 39, 1000 Ljubljana, Slovenia
3 Department of Surface Engineering, Jozef Stefan Institute, Jamova cesta 39, 1000 Ljubljana, Slovenia; janez.kovac@ijs.si (J.K.); gregor.primc@ijs.si (G.P.); ita.junkar@ijs.si (I.J.); alenka.vesel@ijs.si (A.V.)
4 Faculty of Electrical Engineering and Computer Science, University of Maribor, Koroška Cesta 46, 2000 Maribor, Slovenia; denis.donlagic@um.si (D.D); simon.pevec@um.si (S.P.)
5 Plasmadis Ltd, Teslova ulica 30, 1000 Ljubljana, Slovenia
* Correspondence:rok.zaplotnik@ijs.si

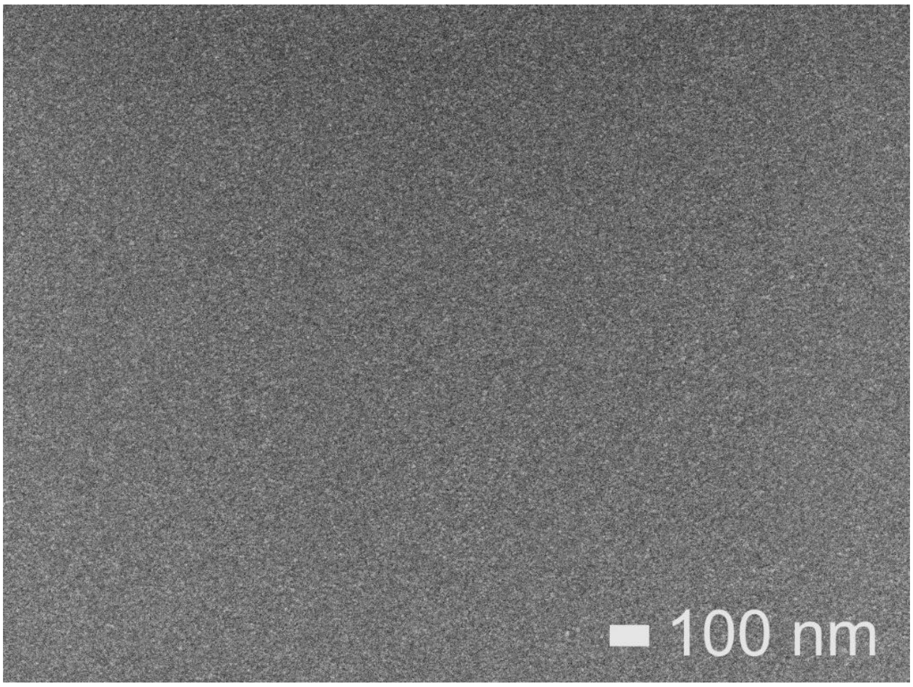


**Figure S1.** SEM image of the HMDSO coating.

© 2020 by the authors. Licensee MDPI, Basel, Switzerland. This article is an open access article distributed under the terms and conditions of the Creative Commons Attribution (CC BY) license (http://creativecommons.org/licenses/by/4.0/).
